# Free Baths for Baltimore

**Published:** 1881-07

**Authors:** 


### EDITORIAL DEPARTMENT.

"If thou hast truth to utter, speak;
And leave the rest to God."

FREE BATHS FOR BALTIMORE.—Whilst the City of Baltimore has advanced with astonishing rapidity in the direction of a great metropolis within the last quarter of a century, and the evidences of a determination to leave nothing undone in the race that looks to the health and comfort of her citizens, are still apparent; she has not yet seen the necessity, or has not been able to undertake what several of her sister cities have already accomplished in the erection of free baths, for the use of the laboring and poorer classes of their citizens. Her recently established *Natatorium* would be credible to any community, and while it is conducted in a manner calculated to meet the approval of all those who are able and willing to avail themselves of the hygienic advantages it is capable of conferring upon them, a large class of her citizens have no proper facilities for bathing within their reach. This latter fact is much to be deplored, as the laboring and poor classes of every community stand in need of such facilities much more even than those in more favorable circumstances.

All physiologists are aware of the great necessity of keeping the skin clean, in order that it may properly perform the important functions required of it in the human organism, at all seasons of the year; and this is particularly important in hot weather among the toiling classes who should be encouraged to practice frequent ablutions, by supplying them with convenient facilities for bathing purposes free of expense or charge! Those who have had much professional experience in warm climates, and during an epidemical visitation, will readily comprehend how great a factor bodily filth, or want of cleanliness, to put it more genteelly, is, in perpetuating such visitations after they have begun their ravages; if indeed, it is not an equally important one in developing the *materies morbi*, upon which their ravages may sometimes depend. Free bathing establishments are investments that probably pay a larger percentage upon the capital which a city may put into them than any other, as they enable the laboring populations to keep themselves in a cleanly state, and consequently in a better condition for resisting morbific influences, thus rendering unavoidable epidemic and endemical visitations more readily amenable to professional treatment, and of shorter duration when they occur. Such establishments are comparatively inexpensive in the outset, and require but little means to keep them running after the outlay necessary in their construction has been made.

